# Endoscopic full-thickness resection of gastric ectopic splenic nodules

**DOI:** 10.1186/s12876-020-01533-3

**Published:** 2020-11-19

**Authors:** Linfu Zheng, Dazhou Li, Wen Wang

**Affiliations:** Department of Gastroenterology, 900th Hospital of PLA, Oriental Hospital Affiliated to Xiamen University, Fujian Medical University, Fuzhou, 350025 China

**Keywords:** Endoscopic full-thickness resection, Ectopic spleen, Gastric submucosal tumor

## Abstract

**Background:**

Ectopic spleen is extremely rare. Most cases are congenital, acquired ectopic spleen may be a consequence of surgery or trauma to the spleen. The ectopic spleen in the gastric wall we reported is even rarer.

**Case presentation:**

We report a 41-year-old female patient, with a past history of splenectomy, who presented with heartburn. Gastroscopy revealed a swelling in the fundus in the stomach. Ultrasonography and computed tomographic examination suggested the possibility of gastrointestinal stromal tumor. We performed endoscopic resection of the mass. Pathological examination of the resected mass showed ectopic spleen.

**Conclusion:**

When a patient with a history of splenectomy presents with a gastric submucosal tumor, ectopic spleen should also be considered in the differential diagnoses. And minimally invasive endoscopic treatment can achieve the purpose of diagnosis and treatment for unobvious submucosal tumors.

## Background

Ectopic spleen is a rare condition that was first described in 1667 by a Dutch doctor, Van Horne [1]. Only about 500 cases of ectopic spleen have been reported so far. The clinical manifestations are nonspecific, and most cases of ectopic spleen are recognized only when complications such as bleeding or abdominal pain occur [2]. In previous reports, the ectopic spleens were mostly located in the pelvis. We present an extremely unusual case of a woman with ectopic splenic nodules in the wall of the stomach.

## Case presentation

**Fig. 1:**
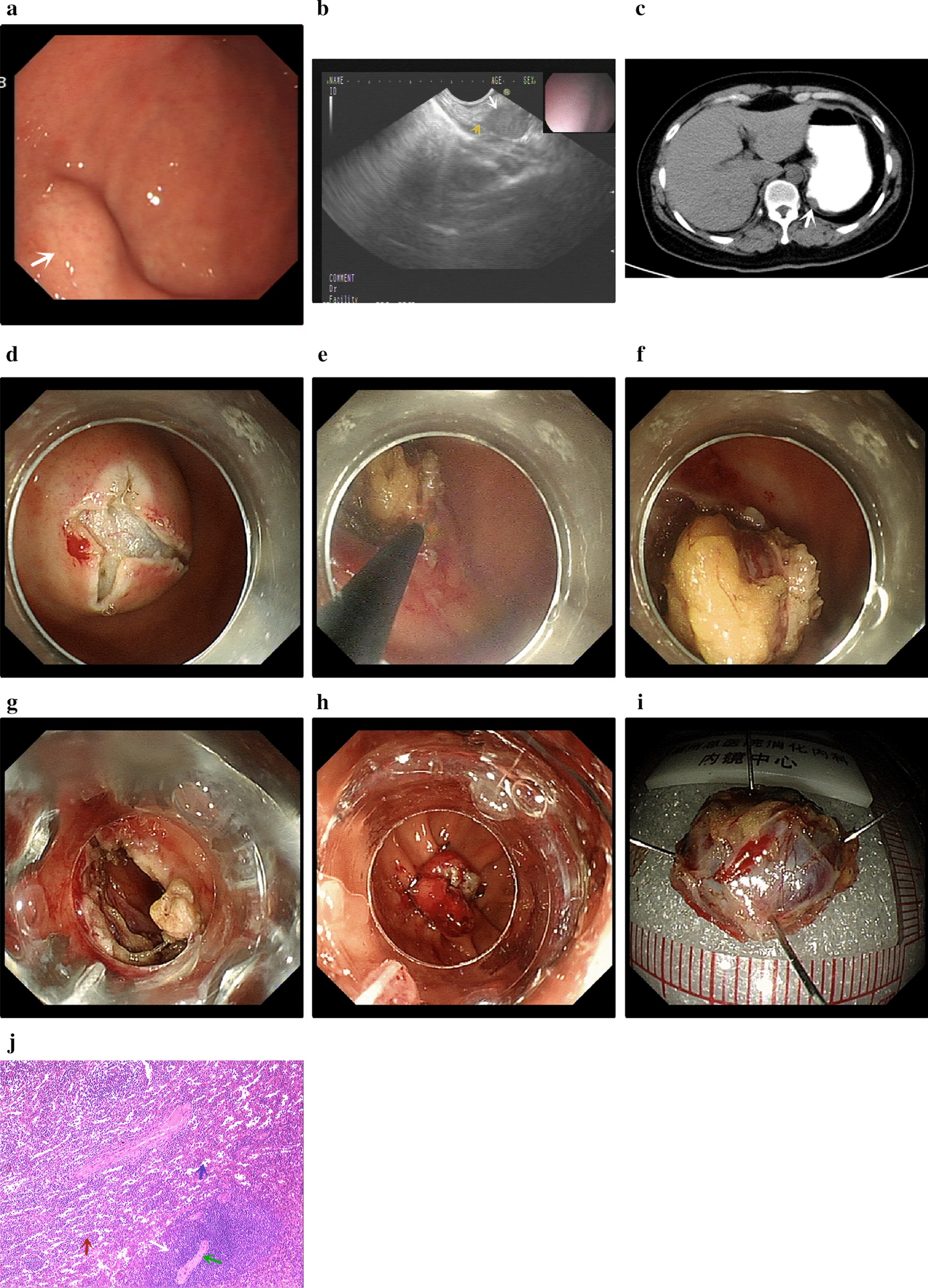
**a** On esophagogastroduodenoscopy a spherical bulge was seen in the fundus of the stomach (white arrow); the overlying mucosa was smooth. **b** Endoscopic ultrasound showed an oval slightly hypoechoic area protruding into the fundus of the stomach without calcification (white arrow); the mass originated from the fourth layer (yellow arrow). **c** Abdominal CT scan suggested the possibility of gastrointestinal stromal tumor (white arrow). **d** The mucosal was incised with Dual-Knife. **e** IT-Knife nano was used to remove the tumor. **f** The tumor was well exposed. **g** After the tumor was completely dissected, the defect in the muscularis was closed by an over-the-scope clip (OTSC). **h** The OTSC closed the wound well. **i.** Gross appearance of the resected tumor. **j** Postoperative pathological examination showed ectopic splenic nodules (hematoxylin and eosin staining, ×40). Red pulp (red arrow), white pulp (white arrow), trabecular vein (blue arrow), and the eccentrically placed central artery (green arrow)

A 41-year-old woman with a history of splenectomy 8 years previously for aplastic anemia was admitted to the 900th Hospital of PLA, Fuzhou, China, with complaints of repeated attacks of heartburn over the last 6 months. The attacks generally occurred after meals. On physical examination, a 13-cm surgical scar was seen on the left lower abdominal wall. No mass was palpable. Laboratory findings revealed red blood cell count 3.59 × 10^12^/L, platelet count 117.0 × 10^9^/L, and normal blood coagulation parameters. Esophagogastroduodenoscopy (EGD) revealed a swelling protruding into the gastric fundus. Endoscopic ultrasonography (EUS) showed an approximately 20.0 mm × 9.6 mm, oval, slightly hypoechoic mass without calcification, originating from the fourth layer, and protruding into the lumen of the stomach; there was no obvious blood flow signal within the mass, and the boundary was clearly seen. Based on these findings, our provisional diagnosis was gastrointestinal stromal tumor (GIST). Computed tomography (CT) of the abdomen and pelvis showed a nodular soft tissue shadow on the greater curvature of the fundus of the stomach; the picture was consistent with GIST. The spleen was absent. We decided to resect the mass, and performed endoscopic full-thickness resection (EFR) under general anesthesia on January 22, 2019. During surgery, a 2.0 cm × 1.8 cm swelling, with smooth overlying mucosa, was seen in the fundus of stomach. A submucosal injection of 1:10,000 adrenaline + methylene blue + sodium hyaluronate was administered at the apex of the lesion, and the lifted mucosa was incised with Dual-Knife (Olympus, Tokyo, Japan). The tumor was found to be located in the muscularis propria. IT-Knife nano (Olympus, Tokyo, Japan) was used to remove the tumor completely. A small perforation was seen at the bottom of the wound, and so an over-the-scope clip (OTSC) was used to close the wound. Postoperative pathological examination of the excised mass showed ectopic splenic nodules. (Fig. 1).

## Discussion and conclusions

Ectopic spleen is very rare, with an incidence of 0.05%-0.5%; there are only sporadic reports in literature [2–4]. According to some scholars, ectopic spleen is common in children under 10 years and in women aged 20–40 years [3–5]. The age of our patient was 41 years, which is consistent with this theory.

Most cases of ectopic spleen are probably congenital [6, 7]. Acquired ectopic spleen is usually a consequence of splenic surgery or trauma. The presence of abundant blood sinuses in the spleen and its strong regenerative ability may lead to implantation and growth of splenic tissue at ectopic sites after trauma [8]. In our patient, for example, it is likely that the ectopic spleen was the result of the previous splenectomy. In addition, our patient had a history of aplastic anemia. Chi has reported an elderly patient with ectopic spleen who also had thalassemia with splenomegaly [3]. Whether patients with hematological diseases are prone to develop ectopic spleen needs to be studied.

The ectopic spleens in previous reports were generally located in the pelvic cavity, with a few in the left upper abdomen [9]. There have been no previous reports of ectopic spleen presenting as a gastric submucosal tumor. In our patient, the initial diagnosis was GIST; ectopic spleen was only diagnosed after pathological examination of the resected mass.

An asymptomatic ectopic spleen, without complications, does not require immediate intervention. However, surgical treatment may be necessary for patients with recurrent abdominal pain, and especially those with suspected torsion, acute infarction or ischemia, rupture, or bleeding of the ectopic spleen [1]. Our patient was offered surgery because she was symptomatic and examination had revealed a tumor protruding into the gastric cavity. She opted for endoscopic treatment. To facilitate complete removal of the tumor, we decided to perform EFR, with OTSC to close the wound. Surgery was uneventful, and there were no postoperative complications; the patient was discharged 5 days after the surgery.

To summarize, in patients with a history of splenectomy, ectopic spleen should be considered in the differential diagnoses of a gastric submucosal tumor. EFR appears to be a safe and effective minimally invasive method for removal of gastric ectopic spleen.

## Data Availability

The materials supporting the conclusions of this article are included in the article.

## Abbreviations

PLA: People’s liberation army
EGD: Esophagogastroduodenoscopy
EUS: Endoscopic ultrasonography
GIST: Gastrointestinal stromal tumor
CT: Computed tomography
EFR: Endoscopic full-thickness resection
OTSC: Over-the-scope clip
